# A Peer Educational Tool to Promote Antimicrobial Stewardship on a University Campus

**DOI:** 10.3390/pharmacy9040199

**Published:** 2021-12-14

**Authors:** Yuman (Yumi) Lee, Nicole Bradley

**Affiliations:** College of Pharmacy and Health Sciences, St. John’s University, 8000 Utopia Parkway, New York, NY 11432, USA; leey2@stjohns.edu

**Keywords:** antimicrobial stewardship, peer education tool, university campus

## Abstract

Antibiotic resistance is a major public health threat. Patient education on the appropriate use of antibiotics is a key component in combating antimicrobial resistance. The purpose of this study was to analyze the utility of an origami fortune teller as a novel peer educational tool in promoting antimicrobial stewardship on a university campus. An origami fortune teller, with various case scenarios to demonstrate key antibiotic principles, was developed and used by peer educators to educate students attending a university wellness fair. The case studies included: antibiotic indications; differentiation between viral vs. bacterial infection; proper use of antibiotics; non-pharmacologic measures to combat infection; and antibiotic resistance. Students were asked to take an assessment pre and post working with the tool. One hundred and forty-three students received education using the novel tool. A significant improvement in the assessment score was observed after education was completed using the novel tool (69.5 vs. 96.6 *p* ≤ 0.05).

## 1. Introduction

Antibiotic resistance is considered a major global public health threat with rates of resistance on the rise. In the United States (U.S.) at least 2.8 million people are infected with an antibiotic resistant infection annually, resulting in over 35,000 deaths [1]. According to the Centers for Disease Control and Prevention (CDC), approximately one third of antibiotic prescriptions in the U.S. are unnecessary [2]. A key driver in these unnecessary prescriptions is patients expecting antibiotic therapy when it is clinically inappropriate. This inappropriate expectation by patients has been shown to significantly impact physician’s decisions to prescribe antibiotics, when they may not be necessary [3].

Combating antibiotic resistance requires high levels of antimicrobial stewardship to ensure the appropriate use of antibiotics. A key component of antimicrobial stewardship is patient education. Public health campaigns targeting public antibiotic education have been shown to have a positive effect on the use of antibiotics [4]. Despite use of public health campaigns, previous studies have demonstrated suboptimal knowledge of antimicrobial stewardship programs in both healthcare students and non-healthcare students.

For example, one study evaluating the understanding of antibiotic stewardship programs among pharmacy students in their final year of training, found that only 21.6% of students had heard about antibiotic stewardship programs [5]. Another study of medical students found that they lacked basic knowledge of antimicrobial resistance [6]. Additionally, a previous survey conducted at our university revealed a knowledge deficit and an inappropriate perception on proper antibiotic use among college students in healthcare programs and non-healthcare programs. The areas identified which required further education included appropriate indications of antibiotics, expectations of antibiotic prescriptions from doctors, and adherence to antibiotics once prescribed. A need was seen to introduce antimicrobial stewardship concepts on a level which college students could grasp [7].

The purpose of this study was to determine the utility of a novel educational tool, an origami fortune teller, by peer educators to promote antimicrobial stewardship on a university campus to healthcare and non-healthcare students.

## 2. Materials and Methods

A novel origami fortune teller educational tool was developed to provide antibiotic education to college students with little to no baseline knowledge in the area. The fortune teller tool was developed by the BUG-OFF (Better Use (of antibiotics) Guards Ourselves, Friends and Family) taskforce which consisted of two Infectious Diseases Pharmacy faculty members and two pharmacy students. The taskforce aimed to mirror key concepts highlighted by the CDC in its patient level antimicrobial stewardship education resources with the development of the educational tool [8]. From this, five key concepts of antimicrobial stewardship were identified to be included as educational points: (1) antibiotics only work against bacterial infections; (2) differentiation between viral vs. bacterial infection; (3) proper use of antibiotics; (4) non-pharmacologic measures to combat infection; (5) antibiotic resistance is a consequence of inappropriate antibiotic use. Patient case scenarios were then developed by the BUG-OFF taskforce to demonstrate these key concepts. Patient case scenarios included:

**Case 1:**  Question: John sits down for lunch. What is the first thing he should do before he digs in?
  Answer: John should wash his hands! Keeping our hands clean is one of the most effective steps we can take to avoid getting sick. The CDC recommends the following steps: 1. Wash your hands; 2. Lather with soap; 3. Scrub for at least 20 s; 4. Rinse; 5. Dry.
**Case 2:**  Question: Jennifer has been diagnosed with the flu. Should she be given an antibiotic to help with her symptoms?
  Answer: No, antibiotics are only effective in treating bacterial infections. Viral infections, like the flu or a cold, cannot be treated with antibiotics. Jennifer should stay home, stay hydrated, and get plenty of rest as antibiotics will not help her.
**Case 3:**  Question: Cathy has been prescribed a 10-day course of antibiotics. On day 6, she starts feeling better. Is it okay for her to stop her regimen?
  Answer: No, Cathy needs to complete the full course of her antibiotics. Failing to take her medication as prescribed can lead to antibiotic resistance. Similarly, taking left over antibiotics can lead to antibiotic resistance.
**Case 4:**  Question: Alex has a runny nose and cough. His mucus is green. Is this a sign of a bacterial infection?
  Answer: No. In general, the color of the mucus is a poor indicator of bacterial or viral infection. Alex is most likely suffering from a cold and antibiotics will not help him.

**Figure 1:**
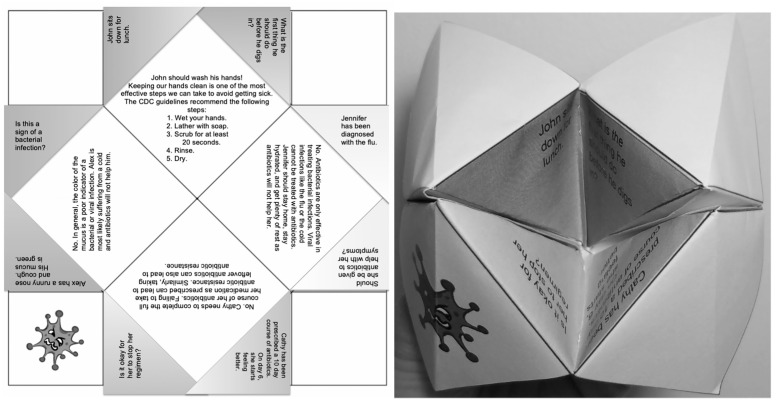
Origami fortune teller educational tool.

The cases were incorporated into an origami fortune teller as shown in Figure 1.

Pharmacy students in their final year of school on Advanced Practice Pharmacy Experience (APPE) rotations were recruited to serve as peer educators to assist with implementing the educational tool at the university wellness fair. A 30 min peer educator training session was held prior to the event. Peer educators were instructed to present the fortune teller and allow the participating student to choose a number and color to select the first case for discussion. The peer educator would guide the discussion and ensure that the participating student arrived at the correct answer, and would continue with the interactive activity until each of the patient cases were discussed.

Study participants were then recruited via convenience sampling of university students attending an on-campus wellness fair. Participants were asked to complete a pre-assessment consisting of 5 items to gauge their baseline understanding of the key antibiotic principles taught using the fortune teller tool. The pre- and post-assessment questions can be found in Table 1. After receiving education using the fortune teller tool, students were asked to complete a post-assessment consisting of the same 5 items.

A sample size of 83 participants was calculated to determine a 20% difference in pre- and post-assessment scores, with an alpha of 0.05 and power of 80%. Data was analyzed using descriptive statistics. The Wilcoxon signed-rank test was used to compare scores on the pre- and post-assessment. This study was reviewed and approved by St. John’s University Institutional Review Board (FWA00009066, 19 November 2019).

## 3. Results

A total of 143 students participated in the antibiotic education session using the origami fortune teller. Approximately 43% of these students were from the College of Pharmacy and Health Sciences (CPHS), and 57% of these students were from non-health science (non-CPHS) colleges in the university. Of the CPHS students, approximately 65% were in the Doctor of Pharmacy Program. Of these, 35% were in their pre-pharmacy years, 27.5% in their first professional year, 7.5% in their second professional year, and 30% in their third professional year.

As seen in Table 2, at the baseline, students scored lowest on Item 1: “antibiotics can help with viral infections like the cold or flu” with only 40.3% correctly identifying this statement as false, and on Item 2: “white, yellow, or green mucus always indicates a bacterial infection” with 41.7% correctly identifying this statement as false. Approximately 88% of students correctly identified Item 3: “it is okay to take leftover antibiotics when I am feeling sick” as false, and Item 4: “proper hand washing includes scrubbing with soap and water for at least 20 s” as true. Nearly 87% correctly identified Item 5: “antibiotic resistance is a public health crisis and caused by the misuse of antibiotics” as true. The overall mean pre-assessment score was 69.5%.

**Figure 2:**
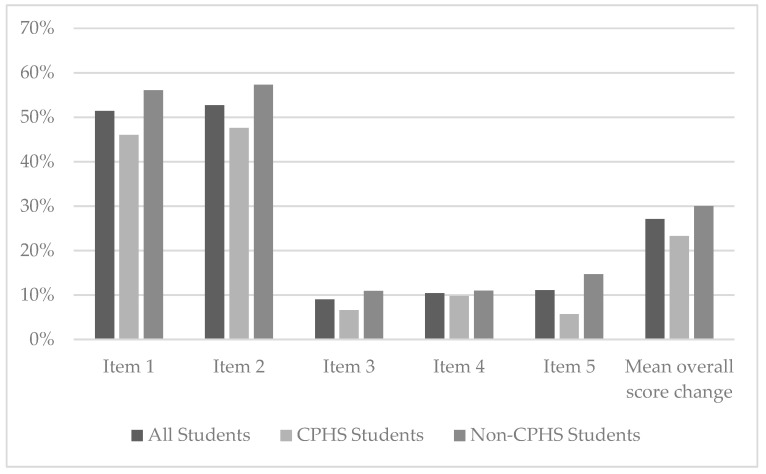
Change in correct responses pre- and post-assessment.

After participating in the antibiotic education session using the origami fortune teller, there was a significant improvement in post-assessment score for each item assessed and in the overall mean post-assessment score compared with the pre-assessment score. Nearly 92% of students correctly answered Item 1, 94.4% answered correctly for Item 2, 97.2% for Item 3, 98.6% for Item 4, and 97.9% for Item 5. The overall mean post-assessment score was 96.6%. The change in pre- and post-assessment correct responses for all participants, CPHS only, and non-CPHS only can be seen in Figure 2.

CPHS students had higher baseline scores on all items, compared with the non-CPHS students. However, CPHS students still experienced a significant improvement in overall score after using the educational tool with a mean pre-score of 75.4% and a post-score of 98.7%. Additionally, significant improvements were observed in pre- and post-assessment scores for Items 1, 2, and 4.

Non-CPHS students also performed significantly better after using the educational tool with mean pre- and post-assessment scores of 65.1% and 95.1%, respectively. Significant improvements in correct responses to Items 1–5 were observed.

## 4. Discussion

Education in antimicrobial stewardship is considered an essential component and a core element to stewardship programs [9]. However, few studies address the impact of providing antibiotic education to the public [4,10]. Oftentimes, public antibiotic education relies on passive teaching tools, such as informational brochures, which rely heavily on the individual to incorporate changes. With passive educational tools, it is very difficult to gauge whether the person received the intended stewardship message [10]. The origami fortune teller provided an interactive learning strategy to improve student antibiotic knowledge, as demonstrated by a significant increase in pre- and post-assessment scores for all items. Use of the interactive fortune teller tool allows for a discussion between the student and educator to ensure that the appropriate message is understood.

To the authors knowledge, no previous studies have detailed the utility of an interactive public stewardship education strategy or use of a novel antibiotic education tool on a university campus targeting both healthcare and non-healthcare students. Although the use of interactive antimicrobial stewardship education has not been studied specifically in a non-healthcare student population, it has been shown to be an effective means of providing antibiotic stewardship education to medical students. One study detailed use of an engaged learning module where medical students participated in an introductory large group discussion on antibiotic over use, then worked in small groups to develop a miniature antibiotic stewardship program [11]. Similar to our findings, this study found that learners were highly engaged and were able to identify and understand common themes of antimicrobial stewardship programs [11]. Another study by Nori and colleagues evaluated the impact of an interactive antimicrobial stewardship curricula for medical students, residents, and fellows. Medical students participated in two training seminars which included small group work and case-based learning modules. Students’ knowledge of general antibiotic concepts improved post seminar [12].

Another benefit of this tool is that it allows for peer education. Studies have shown that peer education provides a positive impact on both the teacher and the learner [13]. This positive impact has been demonstrated across several educational settings including peer teaching as an educational tool in pharmacy schools [14]. In our study, APPE students were able to master key concepts of antibiotic stewardship while teaching others using the fortune teller tool.

Although the CPHS students had higher pre-assessment scores compared with the non-CPHS students, both groups still experienced a significant increase in mean assessment score after using the fortune teller tool. This highlights the versatility of the tool as a means of educating students with some baseline knowledge of antibiotics and those without. Additionally, the tool can easily be expanded to include more patient case scenarios that demonstrate the key principles of antibiotic stewardship.

Limitations of this study include the inability to individually assess the utility of the origami fortune teller apart from the overall peer educational program, the small sample size, lack of tool validation, no long-term follow-up to gauge student retention of the concepts covered, and no comparison to traditional educational tools. Additionally, formal assessment on student engagement and enjoyment was not performed. Anecdotally, verbal positive feedback was received from the students who participated in this learning experience.

## 5. Conclusions

Education is essential to effective antimicrobial stewardship. The novel origami fortune teller tool demonstrated positive trends in increasing student antimicrobial stewardship knowledge. The tool was effective in CPHS and non-CPHS students and offered an opportunity for peer education.

## Figures and Tables

**Table 1:** Pre and Post Assessment Items.

Item 1: Antibiotics can help with viral infections like the cold or the flu. True/False/I Don’t Know Item 2: White, yellow, or green mucus always indicates a bacterial infection. True/False/I Don’t Know Item 3: It is okay to take leftover antibiotics when I am feeling sick. True/False/I Don’t Know Item 4: Proper hand washing includes scrubbing with soap and water for at least 20 s. True/False/I Don’t Know Item 5: Antibiotic resistance is a public health crisis and caused by the misuse of antibiotics. True/False/I Don’t Know

**Table 2:** Correct responses to assessment.

	Pre-Assessment (*n*,%)	Post-Assessment (*n*,%)	Wilcoxon Signed-Rank Test
All Students (N = 143)			
Item 1	58 (40.3)	132 (91.7)	Z = −7.28, *p* < 0.05
Item 2	60 (41.7)	136 (94.4)	Z = −2.80, *p* < 0.05
Item 3	127 (88.2)	140 (97.2)	Z = −2.95, *p* < 0.05
Item 4	127 (88.2)	142 (98.6)	Z = −3.20, *p* < 0.05
Item 5	125 (86.8)	141 (97.9)	Z = −3.31, *p* < 0.05
Mean overall score (%)	69.5	96.6	Z = −8.85, *p* < 0.05
CPHS Students (N = 61)			
Item 1	30 (49.1)	58 (95.1)	Z = −4.32, *p* < 0.05
Item 2	31 (50.8)	60 (98.4)	Z = −4.54, *p* < 0.05
Item 3	57 (93.4)	61 (100)	--
Item 4	55 (90.2)	61 (100)	Z = −2.20, *p* < 0.05
Item 5	57 (94.3)	61(100)	--
Mean overall score (%)	75.4	98.7	Z = −5.59, *p* < 0.05
Non-CPHS Students (N = 82)			
Item 1	28 (34.1)	74 (90.2)	Z = −5.91, *p* < 0.05
Item 2	29 (35.4)	76 (92.7)	Z = −5.84, *p* < 0.05
Item 3	70 (85.4)	79 (96.3)	Z = −2.40, *p* < 0.05
Item 4	72 (87.8)	81 (98.8)	Z = −2.40, *p* < 0.05
Item 5	68 (82.9)	80 (97.6)	Z = −2.82, *p* < 0.05
Mean overall score (%)	65.1	95.1	Z = −6.89, *p* < 0.05
